# Glucocorticoid receptor represses brain-derived neurotrophic factor expression in neuron-like cells

**DOI:** 10.1186/s13041-017-0295-x

**Published:** 2017-04-12

**Authors:** Hui Chen, Marc Lombès, Damien Le Menuet

**Affiliations:** 1 Inserm 1185, Fac Med Paris Sud, Université Paris-Saclay, Le Kremlin-Bicêtre, France; 2 Service d’Endocrinologie et des Maladies de la Reproduction, Assistance Publique-Hôpitaux de Paris, Hôpital de Bicêtre, Le Kremlin Bicêtre, F-94275 France

**Keywords:** Glucocorticoid receptor, Brain-derived neurotrophic factor, Glucocorticoids, Promoters

## Abstract

Brain-derived neurotrophic factor (BDNF) is involved in many functions such as neuronal growth, survival, synaptic plasticity and memorization. Altered expression levels are associated with many pathological situations such as depression, epilepsy, Alzheimer’s, Huntington’s and Parkinson’s diseases. Glucocorticoid receptor (GR) is also crucial for neuron functions, *via* binding of glucocorticoid hormones (GCs). GR actions largely overlap those of BDNF. It has been proposed that GR could be a regulator of BDNF expression, however the molecular mechanisms involved have not been clearly defined yet. Herein, we analyzed the effect of a GC agonist dexamethasone (DEX) on BDNF expression in mouse neuronal primary cultures and in the newly characterized, mouse hippocampal BZ cell line established by targeted oncogenesis. Mouse *Bdnf* gene exhibits a complex genomic structure with 8 untranslated exons (I to VIII) splicing onto one common and unique coding exon IX. We found that DEX significantly downregulated total BDNF mRNA expression by around 30%. Expression of the highly expressed exon IV and VI containing transcripts was also reduced by DEX. The GR antagonist RU486 abolished this effect, which is consistent with specific GR-mediated action. Transient transfection assays allowed us to define a short 275 bp region within exon IV promoter responsible for GR-mediated *Bdnf* repression. Chromatin immunoprecipitation experiments demonstrated GR recruitment onto this fragment, through unidentified transcription factor tethering. Altogether, GR downregulates *Bdnf* expression through direct binding to *Bdnf* regulatory sequences. These findings bring new insights into the crosstalk between GR and BDNF signaling pathways both playing a major role in physiology and pathology of the central nervous system.

**Electronic supplementary material:**

The online version of this article (doi:10.1186/s13041-017-0295-x) contains supplementary material, which is available to authorized users.

## Introduction

The neurotrophin brain-derived neurotrophic factor (BDNF) is a key player in neuronal function. BDNF is highly expressed throughout the brain [1, 2], yet its strongest expression level is found within the hippocampus, a limbic structure of major importance for cognitive functions, such as memorization, learning, behavior, stress, emotions, and mood [3, 4]. In the central nervous system (CNS), BDNF regulates neuronal survival [5], differentiation and growth [6]. Growing evidence indicates that BDNF is also involved in neuronal homeostasis and brain plasticity-related processes such as memory, learning [7, 8] and drug addiction [9], as well as in long term potentiation [10]. Alterations in BDNF expression levels within specific neuron subpopulations have been associated with various pathologies, including depression, epilepsy, Alzheimer’s, Huntington’s and Parkinson’s diseases [11–16]. BDNF mainly functions by binding to its high-affinity receptor, tropomyosin-related kinase B (TrkB) activating several pathways such as MAP kinase, PI3 kinase and Phospholipase C [17]. Rodent *Bdnf* gene exhibits a complex genomic structure comprising of at least 9 exons (I to IX), which are alternatively spliced to generate exon-specific BDNF transcript variants with one common and unique coding exon IX at the 3’ terminal end [18]. Generation of a large set of transcript isoforms is probably of biological significance as in rat hippocampal neuronal cultures, it has been demonstrated that BDNF mRNA variants are differentially distributed in specific dendritic compartments in order to regulate the local availability of BDNF protein [19]. Moreover, BDNF expression was reported to be reduced with aging and associated with a repressed chromatin state on some of its gene regulatory regions [20]. Along this line, epigenetic histone modifications and DNA methylation marks have recently been identified as complex and crucial mechanisms enabling modified expression of various BDNF mRNA isoforms [21]. Altogether, several layers of events driving quantitatively and qualitatively BDNF expression highlight its crucial contribution to CNS function in physiology and pathology [22–24].

Glucocorticoid hormones (GCs) also exert pleiotropic actions on neurons by binding to and activating the glucocorticoid receptor (GR, NR3C1), as well as to the mineralocorticoid receptor (MR, NR3C2) [25, 26]. The latter exhibits a high ligand affinity, and as a consequence it is almost permanently occupied by GCs, while GR is mostly activated under high circulating GC concentrations such as during stress conditions or at the circadian peak of GCs. Both receptors are highly expressed in the hippocampus, acting in balance to regulate various physiological and neurological processes such as stress responses, apoptosis survival and long term potentiation [27]. Interestingly, BDNF activation of TrkB receptors regulates positively GR activity on its target gene expression by phosphorylating two key serine residues on the receptor [28]. Mutating these BDNF-sensitive sites results in the inhibition of the neuroplasticity response to chronic stress [29], unraveling a crosstalk between GC and neurotrophin signaling pathways. On the other hand, regulation of BDNF expression by stress [30] has important consequences on the pathophysiology of mood disorders [31] and in the mechanism of action of antidepressant agents [32]. As exposure to acute or chronic stress triggers a surge of circulating GC concentrations [33, 34], a role of these hormones in modulating BDNF expression has often been suggested [35–41], but most of these reports are based on indirect evidence, and are sometimes contradictory depending on the model and the treatment timeline [42–44]. As a whole, the molecular mechanisms by which GCs regulate BDNF expression are not clearly defined. In the present study, we demonstrated that, upon exposure to the glucocorticoid agonist dexamethasone (DEX), GR directly downregulates *Bdnf* expression, at least in part, by its binding to a specific DNA region upstream of exon IV. Interestingly, this promoter fragment was already characterized as stimulated by synaptic activity in humans and rats [45, 46]. Along with primary cultures of fetal hippocampal neurons (PCN), we used the newly characterized BZ cell line which was previously generated by targeted oncogenesis strategy [47] from a mouse hippocampus and which expresses a high level of both BDNF and GR. Altogether, this work unravels new insights about the repression by GR of *Bdnf* expression, findings that may be of potential physiological importance.

## Methods

### Primary cultures of fetal mouse hippocampal neurons

Pregnant SWISS mice at 18 or 19 days post-fertilization were euthanized by decapitation. Dissection was performed according to a video published in the Journal of Visual Experiments [48]. Hippocampal neurons were isolated and cultured from the embryos using the Pierce Primary Neuron Isolation Kit (Thermo scientific, Courtaboeuf, France) following the manufacturer’s instructions. This kit contained a neuronal media culture supplement (reference: 88286). Cells were typically seeded on culture plates coated with 10 μg/mL poly-D-lysine (Sigma-Aldrich, Lyon, France), at the density of 2.5 × 10^5^ cells/cm^2^ and grown for 9 days at 37 °C in a 5% CO_2_ incubator.

### Cell culture and reagents

BZ and Neuro-2a (N2A, ATCC number: CCL-131) growth medium was composed of DMEM (PAA, Vélizy-Villacoublay, France) containing 10% fetal bovine serum (FBS) (AbCys SA, Paris, France), 1x nonessential amino acids (PAA), 2 mM glutamine (PAA), 100 U/ml penicillin (PAA), 100 μg/ml streptomycin (PAA), 20 mM HEPES (PAA). For BZ differentiation experiments, serum concentration was lowered to 1% for 2 days. N2A differentiation medium was similar to BZ medium but was supplemented with 5 μg/ml insulin (Sigma-Aldrich), 5 μg/ml transferrin (Sigma-Aldrich), 29 nM sodium selenate (Sigma-Aldrich) and 1 μM retinoic acid (Sigma-Aldrich). For hormonal treatment, cells were grown in Dextran Charcoal Coated-treated (DCC) FBS. Dexamethasone (DEX) (Sigma-Aldrich) and RU 486 (RU) (Sigma-Aldrich) were used at the indicated concentrations diluted in ethanol. For RNA stability analysis, 10 μg/ml of 5, 6-Dichlorobenzimidazole-1-β-D-ribofuranoside (DRB) (Sigma-Aldrich) diluted in DMSO was applied.

### Immunocytochemistry

BZ cells cultured in x-well tissue culture chambers (Sarstedt, Numbrecht, Germany) were fixed with PBS containing 4% paraformaldehyde (Sigma-Aldrich) solution for 15 min at room temperature. Cells were permeabilized with 0.5% Triton X-100 (Sigma-Aldrich) for 5 min and blocked by 1% non-fat milk in PBS with 0.1% Tween-20 (PBST) for 1 h at 37 °C. Cells were incubated with anti β-tubulin III antibody TU20 (sc-51670, Santa Cruz, La Jolla, CA, [49]) or with anti-GR antibody M20 (sc-23476, Santa Cruz, [50]) overnight at 4 °C and then with secondary antibody Cy3 (Interchim, Thermo scientific) for 45 min in PBST with 1% milk. After washing, BZ cells were incubated with DAPI (4′,6′-diamidino-2-phenylindole), 1: 1000 in PBST for 2 min and observed with a fluorescence Olympus microscope AX70 (Olympus, Hambourg, Germany).

### RNA extraction and quantitative real-time PCR

Gene expression was quantified by reverse transcription (RT) followed by quantitative PCR (qPCR). Cells were harvested and total RNAs were extracted with Trizol reagent (Invitrogen, Cergy-Pontoise, France) according to the manufacturer’s instructions and their concentrations were determined using a Nanodrop 2000 spectrophotometer (Thermo scientific). RNAs were reverse-transcribed and processed for real-time PCR on an ABI Step One Plus (Applied Biosystems, Courtaboeuf, France). Briefly, 1 μg of total RNA was treated by DNAse I (Invitrogen), then reverse-transcribed with 50 U MultiScribe reverse transcriptase (Applied Biosystems). After 10-fold dilution, 1/40 of the RT reaction was used for qPCR using the Fast SYBR Green PCR master mix (Applied Biosystems). Final primer concentrations were 300 nM for each primer. Primer sequences for each gene are listed in Additional file 1: Table S1. Reaction parameters were 95 °C for 20 s followed by 40 cycles at 95 °C for 3 s and 60 °C for 30 s. For plasmids used for standard curves, amplicons were purified from agarose gels and subcloned into pGEMT-easy plasmid (Promega, Charbonnières, France), then sequenced to confirm the identity of each fragment. Standard curves were generated using serial dilutions of standard plasmids, spanning six orders of magnitude and yielding correlation coefficients more than 0.98 and efficiencies of at least 0.95, in all experiments. Standard and sample values were determined in duplicate from each sample. Relative expression within a given sample was calculated as the ratio: attomol of specific transcripts/attomol of 36B4 transcripts.

### Protein extraction and Western blotting

Protein expression was assessed by Western blotting. Cells were harvested from the culture plates and total cellular proteins were extracted by lysis buffer containing 1% Triton X-100, 1% proteasome inhibitor cocktail (Sigma-Aldrich). Lysates were cleared by centrifugation at 13,000 g for 20 min and protein concentrations were measured using the BC Assay Protein Quantitation Kit (Uptima, Oakland, CA). Total protein lysates (50 μg) were fractionated on 15% SDS-PAGE then transferred to nitrocellulose membranes (GE Healthcare Life Sciences, Vélizy-Villacoublay, France). Membranes were blocked for 1 h in PBST with 2.5% BSA, and then incubated with primary antibodies overnight at 4 °C, rabbit polyclonal anti-BDNF (sc-546, Santa Cruz [51]) at 1:200 and mouse monoclonal anti-α-tubulin (Clone DM1A, Sigma-Aldrich) at 1:10,000. Incubation with secondary antibodies conjugated to infrared fluorophores (goat anti-rabbit IgG Dylight 800 and anti-mouse IgG Dylight 680 at 1:10,000 from Thermo Scientific) was performed for 1 h. An Odyssey infrared imaging system (LI-COR, Bad Homburg, Germany) was used to scan membranes at a wavelength of 680 nm (anti-mouse) or 800 nm (anti-rabbit). Data were analyzed with Image Studio 1.1 software (Li-COR).

### Luciferase assays

pCDNA3-GR plasmid was generated by subcloning human GR (*NR3C1*) into pCDNA3 vector (Invitrogen). *Bdnf* promoter reporter plasmids were constructed by inserting PCR fragments from *Bdnf* promoter regions into PGL4-basic luciferase reporter vector (Promega), see Additional file 1: Table S1 for primer sequences. These fragments, named LP6, SP6, LP4, and SP4, are localized according to the distance to exon VI transcription starting site that was set at +1 (see Fig. 4). Specific genomic sequences were amplified by PCR from mouse genomic DNA and inserted into the PGL4 cloning vector in the *Xho I* and *Hind III* restriction sites using pGEMT easy vector kit (Promega), then transformed in JM109 E.Coli. Plasmids were sequenced to confirm the identity and orientation. The renilla activities driven by the expression vector PRL-TK (Promega) or total protein concentrations were used for normalizing expression.

On the day prior to transfection, N2A cells were plated in 96-well plates at a density of 2 × 10^4^ cells per well in the N2A differentiation medium. Medium was changed for OptiMEM medium (PAA) on the next day, and cells were transfected using Lipofectamine 2000 reagent (Invitrogen). Plasmid concentrations per well were 50 ng PGL4-*Bdnf* promoter vectors, 25 ng pCDNA3-GR or empty vector, 13.3 ng PRL-TK renilla expression vector. Six h post-transfection, OptiMEM medium was replaced by N2A DCC medium. Cells were treated with Ethanol (Veh), DEX and/or RU486 24 h after transfection and then were harvested after another 24 h with Passive Lysis Buffer from the dual luciferase reporter assay system kit (Promega). Lysates were transferred to 96-well clearbottom microplates (Fisher), and luminescence intensities were measured with a TRISTAR LB941 automatic luminometer (Berthold, Thoiry, France) with dual injectors. Normalized values were used for statistical analyses. Each experiment was performed with 8 replicates and repeated at least three times.

### Chromatin immunoprecipitation (ChIP)

ChIP experiments were performed using the Ideal-ChIP for transcription factor kit (Diagenode, Liège, Belgium) following the manufacturer’s protocol. Specifically, 4 × 10^6^ BZ cells per well of 6-well plates grown in 1% DCC medium were treated by vehicle (ethanol), Dex 10^-7^ M or Dex 10^-7^ M together with RU 10^-6^ M for 1 h. Cells were then fixed by a mix of formaldehyde 16% and fixation buffer from the kit (4: 1 ratio), 1/100 volume final for 8 min. ChiP experiments were performed according to Le Billan et al [52]. Chromatin samples (300 μl) were sheared by 12 cycles 30 s ON and 30 s OFF with the Diagenode Bioruptor Pico system and 2.5 μl samples were kept for input measurements. Two hundred and fifty μl of sheared chromatin samples were incubated overnight at 4 °C with 1 μg IgG from the kit as control or 5 μg anti-GR antibody H300 (Santa Cruz sc-8992-X [53]). Chromatin shearing was controlled on a 1.5% agarose gel and typically a smear was visualized ranging from 100 to 500 bp. qPCR amplifications of eluted DNA were performed with primers encompassing a short upstream sequence of exon IV, or on *Per1* and *Ucp1* promoters (Additional file 1: Table S1 for primer sequences). Raw data are expressed as percentage of inputs, according to the Percent of Input Method, ChIP analysis; Thermo Fischer Scientific.

### Immunoprecipitation

Immunoprecipitations were performed using 30 μl protein A-coated magnetic beads (Diagenode). Beads were washed 3 times with C1 buffer of the High Cell ChIP kit (Diagenode) and incubated for 4 h with anti-GR antibody H300 (Santa Cruz) at 4 °C with protease inhibitors and 0.2% BSA. Then, 350 μg of proteins from BZ cell lysates were incubated overnight with antibody-coated beads. The following day, beads were washed 3 times with buffer C1 and once with buffer W1 (High Cell ChIP kit), and immunoprecipitated proteins were eluted from the beads with 20 μl of Laemmli buffer at 95 °C for 5 min and loaded on a 10% SDS-PAGE gel for Western blotting. Membranes were hybridized with anti-GR antibody M20 (sc-23476, Santa Cruz, [54].

### Targeted mutagenesis

Targeted mutations of *Bdnf* promoter construct plasmids were performed using the Quickchange II XL kit (Agilent Technologies, Les Ulis, France) following the manufacturer instructions. Briefly, 10 ng of SP4 luciferase plasmid was amplified by PCR with sense and antisense mutated primers for 18 cycles. PCR reactions were transformed in XL1 blue ultracompetent E. Coli strain (Agilent, les Ulis, France) on LB Agar Ampicillin petri dishes. Bacterial colonies were picked up. Plasmid DNA were extracted and sequenced by Eurofins (Ivry sur Seine, France) to check for the introduction of the mutation and sequence integrity. Primers for mutagenesis are available in Additional file 1: Table S1. Plasmids were transfected in N2A cells as stated for the luciferase assay.

### Statistical analyses

Results are expressed as mean ± SEM of at least six samples for each condition unless stated otherwise. Statistical analyses were performed using nonparametric Mann-Whitney U-tests, unless stated otherwise, using Prism 5 (GraphPad Software, Inc., San Diego, CA).

## Results

### GR represses *Bdnf* expression in primary mouse hippocampal cultures

**Fig. 1:**
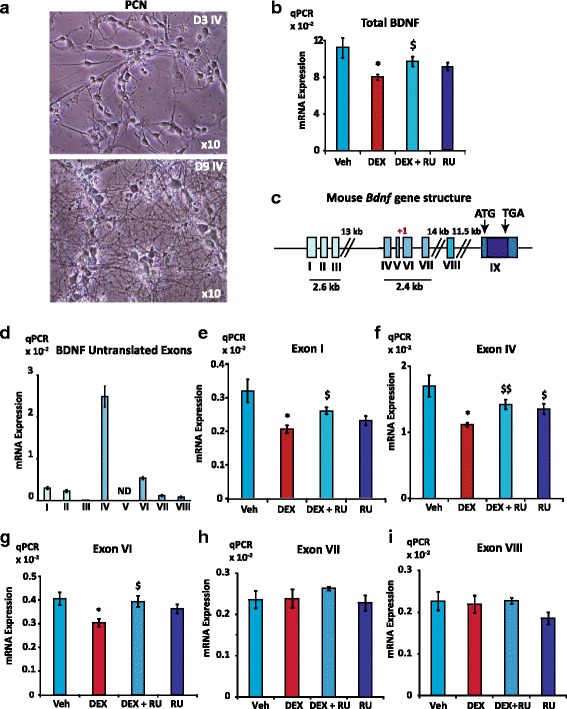
GR inhibits *Bdnf* expression in 9-day PCN. **a**, Bright field microscopy showed that PCN developed a morphology with multiple cellular processes on Day 3 in vitro (D3 IV, upper panel), while a denser neuronal network was visible on Day 9 (D9 IV); magnification x10. **b**, Total BDNF mRNA expression was significantly repressed by DEX. Relative expression of total BDNF mRNA assessed by qPCR on exon IX under vehicle, (Veh, ethanol) dexamethasone (DEX; 10^-7^ M) or DEX and GR antagonist RU486 (RU; 10^-6^ M) treatments (1 h). Results are expressed as attomol of BDNF mRNA/attomol of 36B4 mRNA (*n* = 6, Mean ± SEM); * Veh *vs* DEX, *P* < 0.05; $ DEX *vs* DEX + RU, *P* < 0.05, Mann Whitney U-tests. **c**, Schematic representation of mouse *Bdnf* gene according to Aid et al [18]. Eight untranslated exons (I to VIII) splice onto one common coding exon IX, with two regulatory regions that drive the expression of mainly 2 clusters of untranslated exons (I, II, III and IV, V, VI, respectively). **d**, Relative expression of *Bdnf* untranslated exons in 9-day PCN (*n* = 6, Mean ± SEM) as measured by RT-qPCR. Exons IV and VI containing transcripts are the most expressed isoforms, followed by that of exon I containing mRNA. ND: non determined. **e**, **f**, **g**, **h**, **i**, Expression of exon I, IV, and VI containing transcripts was downregulated by DEX while expression of exon VII and VIII containing transcripts was not modified by DEX treatment. qPCR analyses of exon I, IV VI, VII and VIII mRNA levels in the same samples than in (**a**), Mean ± SEM; * (*P* < 0.05) *vs* Veh; $*P* < 0.05; $$ *P* < 0.01 *vs* DEX; Mann Whitney U-tests

To determine the effect of GCs on *Bdnf* expression in neurons, we used day 9 primary cultures of mouse hippocampal neurons (PCN), a model of high physiological relevance that expresses both BDNF and GR (Additional file 2: Figure S1a and c). Bright field microscopy at day 3 in vitro (D3 IV, Fig. 1a upper panel) already showed a nearly homogenous neuronal culture that developed in a dense neuronal network at day 9 (D9 IV, Fig. 1a, lower panel). Neuronal marker MAP2 transcripts expression measured by qPCR was in the same range in PCN than in mouse brain while glial marker GFAP expression, assessed as an estimation of astrocyte contamination displayed a more than two hundred times lower expression in PCN culture than in mouse brain (Additional file 2: Figure S1b and d), indicating a high neuronal enrichment in PCN culture. Additionally, GR mRNA and protein were clearly expressed in D9 IV cultures, as shown in qPCR (Additional file 2: Figure S1c) and in Western blot (Additional file 2: Figure S1e). One h treatment with dexamethasone (DEX), a synthetic GR agonist (10^-6^ M), was sufficient to significantly repress by 30% total BDNF mRNA expression (Fig. 1b), measured by qPCR on the common exon IX abundance. This inhibition was fully reversed by co-treatment with the GR antagonist RU486 (RU). These results were consistent with a previous study performed on rat PCN [42]. These experiments were performed using a neuronal media supplement (Pierce neuronal isolation kit, reference 88286) that was required for the survival of PCN and that did contain GC. We were unable to maintain the cells in culture in the absence of the supplement even for a few h. Of note, the culture medium with the supplement contains a substantial concentration of corticosterone (~2.10^-8^ M), as measured by LC/MS-MS (data not shown), that is sufficient to partially bind and activate GR.

In order to better understand the mechanisms by which GCs decrease neuronal *Bdnf* expression, it is worth noting that mouse *Bdnf* gene exhibits a complex genomic structure with 8 untranslated exons (I to VIII) that splice onto the common coding exon IX and two clusters of promoters upstream of I, II, III and IV, V, VI, respectively (Fig. 1c) [18, 55]. We determined the relative expression of the untranslated exon-containing transcripts by RT-qPCR and found that exon IV containing mRNA isoform was by far the most expressed one followed by exon VI and I containing isoforms (Fig. 1d). Expression of all of these 3 exon-containing messengers was repressed by DEX while RU suppressed this effect (Fig. 1e, f and g). Conversely, DEX did not exert any effect on exons VII and VIII containing transcripts (Fig. 1h and I), highlighting an exon-specific regulation of GR on *Bdnf* gene. Altogether, these data showed that GR was able to repress BDNF mRNA expression in PCN by downregulating specific isoforms even in the presence of noticeable amounts of GCs in the medium.

### The BZ cell line is a glucocorticoid responsive model expressing BDNF

**Fig. 2:**
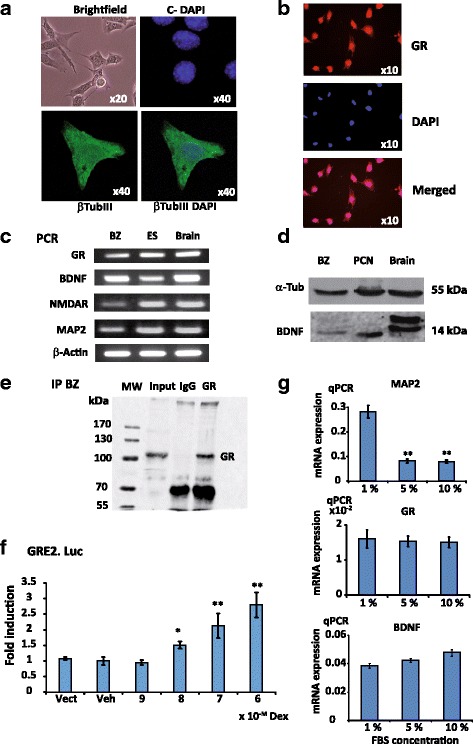
Characterization of the BZ cell line. BZ cells are cultured in DMEM supplemented with 1% FBS unless stated otherwise. **a**, BZ cells present with neuronal features. BZ cells display an elongated morphology harboring multiple short cellular processes, brightfield microscopy (magnification x20, upper left panel). Immunolabeling images (x40) with negative control secondary antibody and DAPI counterstaining (upper right panel), β-tubulin III (lower left panel) and merged DAPI and β-tubulin III (lower right panel). **b**, GR is expressed in BZ cells. Immunocytochemistry of GR staining on BZ cells grown in 10% FBS, showing a homogenous expression of GR in this cell line. GR, upper panel, DAPI, middle panel, Merged, lower panel (**c**), Analytical RT-PCR of GR, BDNF, NMDA receptor ζ (NMDAR) and MAP2 mRNA expression (30 cycles of amplification with DreamTaq (Thermo Scientific)) on BZ cells compared with neurons derived from mouse embryonic stem cells (ES) and mouse brain. **d**, Western Blot on BDNF protein in BZ cells, primary cultures of mouse hippocampal neurons (PCN), and mouse brain, showing that BDNF protein was clearly detected in all of the 3 samples. **e**, Immunoprecipitation in BZ cell lysates with anti-GR antibody, further demonstrating GR expression, MW: molecular weight markers in kDa, IgG: rabbit immunoglobulins, GR band is around 105 kDa. **f**, DEX was able to transactivate GRE2.Luc, indicating the presence of a functional GR in BZ cells. Transfection of BZ with a PGL3-Gre2.Luc plasmid under DEX dose responses (10^-9^ to 10^-6^ M, *n* = 8, Mean ± SEM); Vect, GRE2.Luc; Veh set at 1; * *P* < 0.05, ** *P* < 0.01, Mann Whitney U-tests. **g**, Increased expression of neuronal marker MAP2 transcripts at low FBS (1%) concentration. MAP2 (higher panel), GR (middle panel) and BDNF (lower panel) mRNA expression in BZ cells under various FBS concentrations from 1 to 10%; *n* = 6, Mean ± SEM; ** *P* < 0.01 *vs* 1% condition, Mann Whitney U- tests

In PCN, the presence of corticosterone in the culture supplement was likely able to interfere with investigation on GR action on BDNF expression. Nonetheless, the BZ cell line previously generated from a mouse hippocampus by targeted oncogenesis [56] displayed several interesting characteristics to study this issue. BZ cells appeared in light microscopy as slightly elongated and connected by short processes when grown in 1% fetal bovine serum (FBS, Fig. 2a, upper left panel). Immunolabeling revealed the presence of the β-tubulin III neuronal marker in BZ cells (Fig. 2a, upper right panel and lower panels), indicating they present with some neuron-like features. Moreover, GR was readily detected by immunocytochemistry as a nucleocytoplasmic labeling in BZ cells (Fig. 2b). As shown by the DAPI counterstaining, GR expression is expressed in all BZ cells. In addition, expression of *Gr*, *Bdnf* as well as the neuronal marker *Map2* and the *NmdaR*ζ1 (*Grin1*) isoform was observed in BZ cells as measured by RT-PCR (Fig. 2c), and compared to embryonic stem cell derived neurons (ES) and mouse brain. Quantification by qPCR of BDNF and GR mRNAs showed that their relative levels were in the same range in BZ cells and PCN (Additional file 2: Figure S1a and c), although lower when compared to those measured in the brain. While BZ cell expression of *Map2* neuronal marker was comparatively lower than the one measured in PCN and brain, it was similar to that found in ES derived-neuron cultures (Additional file 2: Figure S1b). It is worth noting that the main BDNF receptor TrKB was not expressed in BZ cells (data not shown), thus any observed effect on BDNF expression is not due to a negative feedback loop resulting from TrkB receptor activation. BDNF protein was clearly detected by Western blotting on lysates from BZ cells and PCN as well as mouse brain (Fig. 2d). GR expression in BZ cells was further demonstrated by immunoprecipitation assays as revealed by the ~ 100 kDa molecular mass band observed in both input and GR immunoprecipitated complexes lanes (Fig. 2e). BZ cells were transfected with a plasmid containing two glucocorticoid response elements fused with the luciferase gene (GRE2.Luc). Although transfection efficiency was low, DEX treatment was able to transactivate GRE2.Luc starting from a dose of 10^-8^ M indicating that a functional GR is expressed in BZ cells (Fig. 2f). We optimized the BZ cell culture conditions by lowering fetal bovine serum concentration from 10 to 1% for 48 h and showed that this experimental condition increased the expression of the MAP2 neuronal marker mRNAs (Fig. 2g). As BDNF and GR transcripts expression are identical between all culture conditions, this effect is probably specific. Thus, BZ cells were routinely expanded using 10% FBS, plated at 12,500 cells/cm^2^ and grown in low serum concentration (1%) 48 h before hormonal treatments. Altogether, the BZ cell line of hippocampal origin exhibits neuronal features and expresses substantial amounts of both GR and BDNF at the messenger and protein levels, enabling to investigate the impact of GR activation on *Bdnf* expression.

### GR represses *Bdnf* expression in BZ cells

**Fig. 3:**
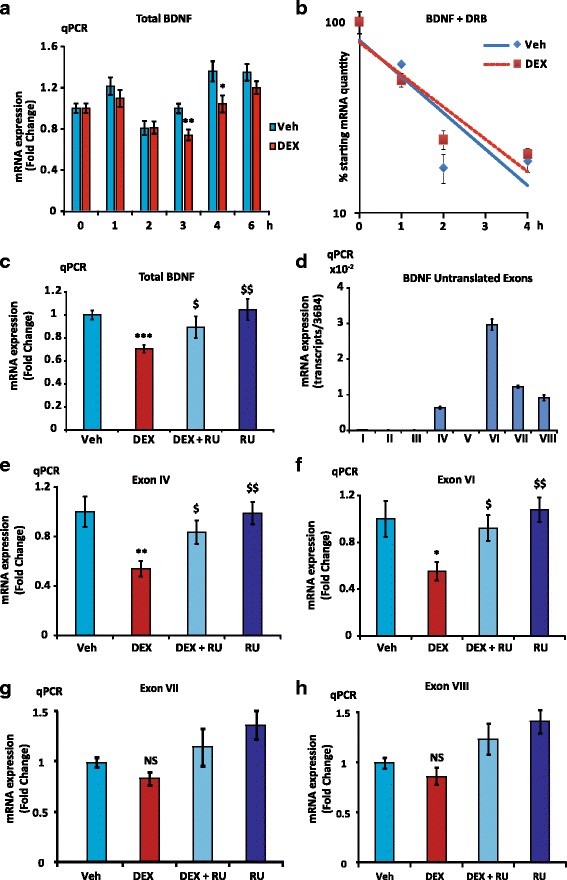
GR downregulates BDNF expression in BZ cells. **a**, The total BDNF transcript abundance was decreased by 30% after 3 and 4 h DEX treatment. Time course of total BDNF transcript expression under 10^-7^ M DEX or vehicle treatment, time 0 is set at 1, n = 12, Mean ± SEM; * *P* < 0.05, ** *P* < 0.01*vs* Veh, Mann Whitney U-tests. **b**, Repression of BDNF mRNA expression is transcriptional. Time course assessment of total BDNF mRNA expression under DRB in the presence or absence of DEX. Note the logarithmic scale of the y-axis. Total BDNF transcript half-life time was estimated at approximately 2 h. The corresponding slopes were not significantly different with ANCOVA test (*P* = 0.14) (**c**), GR effect is inhibited by RU. RT-qPCR on common exon IX, 3 h treatment with DEX 10^-7^ M and/or RU 10^-6^ M (*n* = 12, Mean ± SEM); Veh *vs* DEX ***P < 0.001, RU and DEX + RU *vs* DEX, $ *P* < 0.05, $$ *P* < 0.01. **d**, Relative expression of BDNF untranslated exons containing mRNA in 4-day BZ cultures (*n* = 6, Mean ± SEM) as measured by RT-qPCR. The highest expressed isoform in BZ cells is exon VI-containing transcript, followed by exon VII and VIII, then exon IV, while exon I mRNA expression was negligible. **e**, **f**, **g**, **h**, GR specifically repressed exon IV and VI expression but was ineffective on exon VII and VIII expression. qPCR on exon IV, VI, VII and VIII (*n* = 12, Mean ± SEM) containing transcript levels in the same samples than in (**c**); Veh set at 1, * (*P* < 0.05), and ** (*P* < 0.001) *vs* Veh; $ (*P* < 0.05) and $$ (*P* < 0.01) *vs* Dex, Mann Whitney U-tests

The effect of DEX on total BDNF mRNA expression was analyzed on time course experiments, using a 10-fold lower concentration (10^-7^ M) than in PCN. DEX significantly decreased total BDNF transcript abundance by 30% after 3 and 4 h treatment (Fig. 3a). At this dose, no effect was observed on PCN probably because of the presence of corticosterone in the culture medium (data not shown). Parallel measurements of the GR target gene *Sgk1* transcript levels revealed, as expected, a strong induction (3 to 5 fold) already detected after 1 h stimulation, thus validating GC treatment effectiveness, and the BZ cell ability to respond to GCs (Additional file 3: Figure S2a). In the presence of the transcription inhibitor DRB, there was no difference in the rate of BDNF mRNA turnover between Veh and DEX, suggesting that the DEX-induced reduction of BDNF transcript levels was related to transcriptional repression rather than RNA decay mechanisms (Fig. 3b). These findings also demonstrated that the half-life time of BDNF transcripts in BZ cells was evaluated at around 2 h, which is consistent with a rapid detection of BDNF mRNA level diminution upon GC exposure. Co-treatment with RU for 3 h abolished the effect of DEX on *Bdnf* expression, demonstrating that DEX repression is indeed mediated by GR (Fig. 3c). As a control, under the same experimental conditions, *Sgk1* activation by DEX was fully repressed by addition of RU (Additional file 3: Figure S2b). The relative expression of *Bdnf* untranslated exons in BZ cells was somehow different from the one observed in PCN since exon VI-containing mRNA represented the highest expressed isoform in BZ cells, followed by exon VII and VIII then exon IV mRNA while the expression of exon I-containing transcript was negligible (Fig. 3d). Examination of exon IV and VI isoform expression after 3 h DEX treatment showed a similar pattern of repression than with total BDNF transcripts, which was fully prevented by coincubation with RU (Fig. 3e and f). Interestingly, DEX-induced repression was stronger with isoform IV and VI (around 50%) than for total BDNF transcripts. Likewise in PCN, there was no effect of DEX on exon VII and VIII (Fig. 3g and h), indicating once again the specificity of the DEX-repressive action on exon IV and VI expression. These findings suggested a GR mediated effect through a repressive mechanism on the promoter regions upstream of exon IV and/or VI (see Fig. 1c).

### GR represses *Bdnf* expression by acting upstream of exon IV

**Fig. 4:**
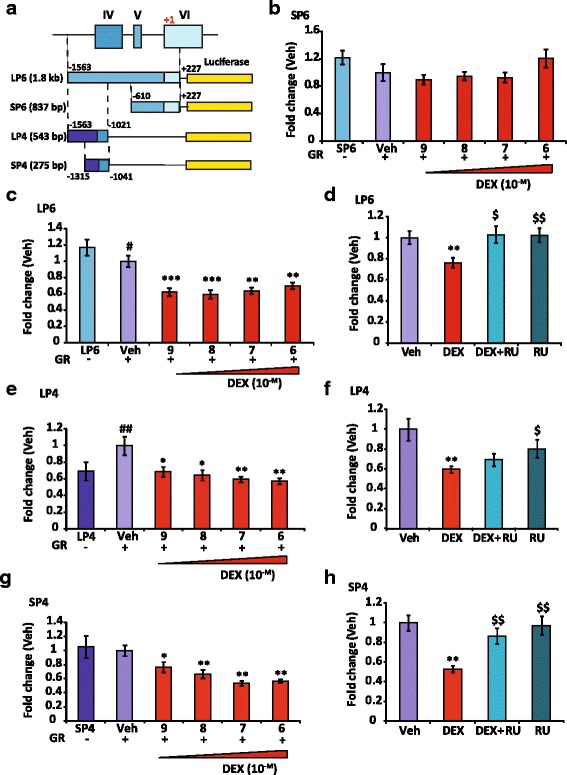
GR-dependent inhibitory effects on BDNF promoter 4 activity relies on a short sequence upstream of exon IV. **a**, Map of transfected constructs, LP6, SP6, LP4 and SP4 regions. These sequences were subcloned into PGL4 luciferase plasmid, setting the first base of exon VI at +1: a long (LP6, 1.8 kb, -1563, +227, containing exon IV, V and part of VI) and three short regions (SP6, 837 bp, -610, +227, partly encompassing exon VI; LP4, 543 bp, -1563, -1021; SP4, 275 bp, -1315, -1041; LP4 and SP4 are upstream of exon IV and include its transcription start site). **b**, DEX dose responses (Veh, Ethanol for 24 h) of SP6 transfected in N2A cells, co-transfected with GR expression vector and Renilla plasmid for normalization. Results are expressed as fold change of the luciferase/Renilla value of SP6 transfected alone. No significance was observed whatsoever (*n* = 16), showing SP6-driven activities were not modified by DEX. **c**, Same as in (**b**) with LP6 construct, Veh condition is set at 1; *n* = 16, Mean ± SEM; # p < 0.05, LP6 *vs* Veh; **P < 0.01, ***P < 0.005, DEX *vs* Veh; Mann Whitney U-tests. Luciferase activities driven by LP6 were already decreased by DEX at a low concentration. **d**, Transfection of LP6 in N2A cells, 24 h treatment with Veh, DEX (10^-7^M), RU (10^-6^M) and both; n = 16, Mean ± SEM; $ *p* < 0.05, $$ *p* < 0.01, vs DEX; Mann Whitney U-tests. The DEX repression is inhibited by RU. **e**, DEX dose responses of LP4 transfected in N2A cells. Results are expressed as fold change of the luciferase/protein value of LP4 transfected alone. Veh condition is set at 1; *n* = 8; ## *P* < 0.01, LP4 vs Veh;* *P* < 0.05, ** *P* < 0.01, DEX *vs* Veh; Mann Whitney U-tests. DEX reduced the LP4 activities in a dose-response manner. **f**, Transfection of LP4 construct plasmid in N2A cells, 24 h treatment with Veh, DEX (10^-7^M), RU (10^-6^M) or both; *n* = 16, Mean ± SEM; *P < 0.05 *vs* Veh; $ *p* < 0.05, *vs* DEX; Mann Whitney U-tests. The DEX effect was not reversed by RU. **g**, Same as in (**e**) with SP4 construct. DEX *vs* Veh; * *P* < 0.05 and ** *P* < 0.01. DEX repressed SP4 activity. **h**, SP4 fragment is repressed by GR. Same as in (**f**) with SP4 construct; ** *P* < 0.01 *vs* Veh; $$ *P* < 0.01 vs DEX

Exons IV and VI are in a close vicinity on mous*e Bdnf* gene, located in a ~ 1.5 kbp sequence (Fig. 4a). To clarify the mechanisms by which activated GR represses neuronal *Bdnf* expression, we generated several *Bdnf* promoter luciferase constructs including four fragments of this region, LP6 (1.8 kb, -1563; +227) and SP6 (837 bp, -610; +227) flanking exon VI, LP4 (543 bp, -1593; -1021) and SP4 (275 bp -1315; -1041) flanking exon IV. The first base-pair of exon VI was arbitrarily set at +1 for the sequence numbering (see Fig. 4a). The activities of these fragments inserted upstream of the reporter luciferase gene were investigated for their responses to GCs using luciferase assays in the neuroblastoma tumor cell line N2A. This model has been preferred to BZ cells given that these latter cells exhibit a very low transfection efficiency. DEX dose response curves (10^-9^ to 10^-6^ M) were generated with the four constructs while cotransfecting a GR expression vector. Whereas the luciferase activities driven by the SP6 construct were not modified by DEX whatever the GC dose (Fig. 4b), LP6-driven activities were significantly reduced even at the lowest DEX concentration (10^-9^ M) (Fig. 4c). Co-treatment with RU abolished DEX inhibitory effect, stressing that this repression was mediated by GR (Fig. 4d). To determine the location of the regulatory regions on which DEX-activated GR affected *Bdnf* expression, we narrowed down our promoter analyses by focusing on two shorter DNA fragments, LP4 and SP4, upstream of exon IV (see Fig. 4a) since DEX did not regulate SP6 activity. Luciferase activities driven by both the two shorter fragments were significantly reduced by GR with DEX compared with vehicle, likewise what was observed with the LP6 fragment, in a dose-response manner (Fig. 4e and g). While DEX was able to repress LP4 construct, (Fig. 4f), significant antagonist effect of RU was observed with the SP4 construct (Fig. 4h) indicating that activated GR may act directly or indirectly on this short 275 bp region upstream of exon IV in an inhibitory fashion.

### AP1 and CRE response elements do not mediate GR transrepression

**Fig. 5:**
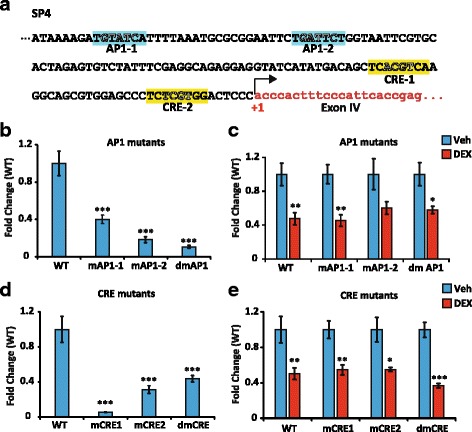
AP1 and CRE response elements do not mediate GR transrepression. **a**, Sequence analysis of a short fragment upstream of exon IV, and included in SP4 sequence that contains two potential AP1 binding sites, TGTATCA and TGATTCT, as well as two potential cAMP Response-Elements (CRE): CRE-1 (for CREB1, TCACGTCA) and CRE-2 (for CREB3l2, TCTCGTGG) according to the Jaspar database; +1 is the transcription start site of exon IV. **b**, Luciferase activity of WT and AP1 mutants. mAP1-1: construct with AP1-1 deletion, mAP1-2, construct with deletion of AP1-2 sequence, dmAP1, deletion of both sites. Deleted based are shown in *white* lettering in Fig. 5a. Basal transcriptional activity driven by the WT sequence was arbitrarily set at 1, Means ± SEM; *** *P* < 0.005; Mann Whitney U-tests, *n* = 8. **c**, Luciferase activities of promoter mutants with AP1 deletions were still repressed by DEX. * *P* < 0.05 DEX (*red*) vs vehicle (*blue*). Mean ± SEM; ** *P* < 0.01, DEX vs Veh of the same construct. Mann Whitney U-tests, *n* = 8; all Veh conditions were set at 1. **d**, Luciferase activity of WT and CRE mutants. mCRE-1: construct with CRE1-1 deletion, mCRE-2, construct with deletion of CRE-2 sequence, dmCRE, deletion of both sites. Deleted based are shown in *white lettering* in Fig. 5a. Basal transcriptional activity driven by the WT sequence was arbitrarily set at 1, *** *P* < 0.005; Mann Whitney U-tests, *n* = 8. **e**, Luciferase activities of promoter mutants with CRE deletions were still repressed by DEX. mCRE-1: construct with CRE-1 deletion, mCRE-2, construct with deletion of CRE1-2 sequence, dmCRE, deletion of both sites. * *P* < 0.05 DEX (*red*) *vs* vehicle (*blue*). Means ± SEM; ** *P* < 0.01, DEX *vs* Veh for the same construct; *** *P* < 0.005, DEX *vs* Veh for the same construct, Mann Whitney U-tests, *n* = 8; all Veh conditions were set at 1

To investigate the molecular mechanisms of the glucocorticoid-dependent repression, we analyzed *in silico* the GR-sensitive SP4 sequence (275 bp; -188, +87 from exon IV transcription start site) using Jaspar database (URL: http://jaspar.genereg.net/cgi-bin/jaspar_db.pl) but were not able to identify any potential GR response elements (GRE). In contrast, we found two Jun/Fos response elements, comprising two potential AP1 binding sites (positions -127 and -101), as well as two cAMP response elements (CRE), for CREB1 (position -38) and CREB3l2 (position -14), see Fig. 5a. Given that GR is able to repress SP4 sequence transactivation ability (see Fig. 4h), it was tempting to speculate that GR effect was the result of a transrepression mediated by its interaction with AP1 and/or CRE binding transcription factors as it has been previously proposed [57]. Of interest, in humans and rats, these CRE response elements, which are evolutionary conserved were characterized as positively regulated by synaptic activity [46], while constructs harboring the AP1 sites were not sensitive to a previously described BDNF-positive feedback loop [58]. To test this hypothesis, deletion mutants of AP1 (mAP1-1 and mAp1-2) and CRE (mCRE1 and mCRE2) binding sites were generated from SP4-luciferase plasmid, as well as double mutants with deletion of the two potential AP1 binding sites (dmAP1) or the two CRE binding sites (dmCRE) sites (Fig. 5a, the deleted bases are indicated in white lettering). All AP1 and CRE mutants, as well as the double mutants showed a strong reduction of their basal transcriptional activities in N2A cells compared to the WT plasmid indicating they are functional sequences in SP4 construct (Fig. 5b and d). The transcriptional response to DEX was measured setting the vehicle condition value at 1 for each construct (Fig. 5c and e). For all these mutants, as well as for AP1 and CRE double mutants (dmAP1 and dmCRE), DEX treatment still led to the repression of their transcriptional activities in a similar fashion than with the SP4 wild type fragment. In the absence of classical GRE and other response elements for transcription factors known to interact with GR in the SP4 sequence, these data excluded the possibility of an interaction between GR and AP1 and/or CRE response elements to transrepress *Bdnf* expression in these neuron-like cells.

### GR binds upstream of exon IV upon DEX treatment

**Fig. 6:**
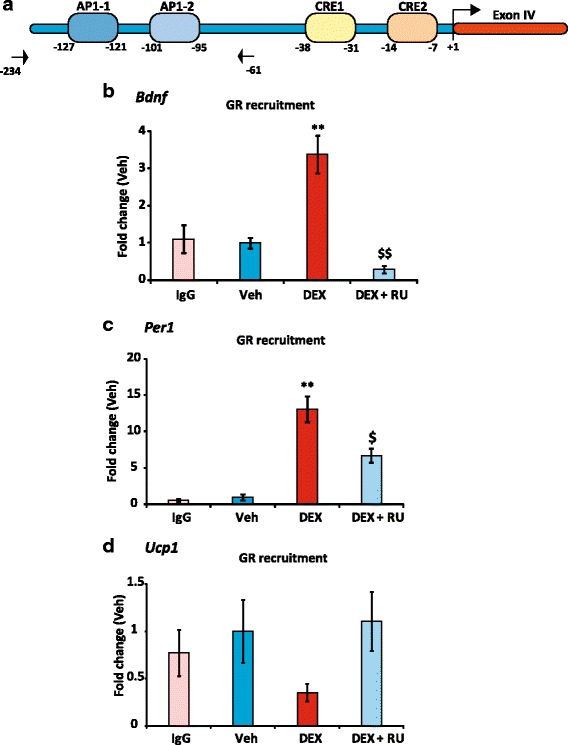
GR recruitment on *Bdnf* DNA sequence. **a**, Schematic representation of a short fragment upstream of exon IV that contains the two potential AP1 binding sites, AP1-1 and AP1-2 as well as two potential cAMP Response-Elements CRE-1 and CRE-2 according to the Jaspar database. Exon IV transcription start site is set at +1. Primers for ChIP are indicated by arrows. **b**, ChIP-qPCR results showing GR recruitment upstream of exon IV region in BZ cells after treatment with DEX (10^-7^ M) or DEX together with RU (10^-6^ M) for 1 h. IgG: ChIP-qPCR with IgG negative control antibodies on the same sequence. Results of 3 different experiments are pooled setting the vehicle mean value at 1 (*n* = 6, Mean ± SEM), and showed as fold change of the percentage of input value of Veh.** *P* < 0.01, DEX *vs* Veh; $$ *P* < 0.01, DEX + RU *vs* DEX; Mann Whitney U-tests. **c**, ChIP-qPCR results (same samples than in B) of GR recruitment on *Per1* gene regulatory sequence used as positive control in BZ cells under DEX or DEX + RU treatment for 1 h. ** *P* < 0.01, DEX *vs* Veh. $ *P* < 0.05, DEX + RU *vs* DEX. **d**, same as in (**c**) with GR recruitment on *Ucp1* gene used as negative control. Primers for genomic PCR amplification are listed in Additional file 1: Table S1

Even if no specific DNA response element was identified for DEX responses, transfection data strongly suggest a direct involvement of GR to repress SP4 transcriptional activity. To determine whether GR does bind to *Bdnf* SP4 promoter fragment, chromatin immunoprecipitation (ChIP) experiments were undertaken in BZ cells using specific primers (see Additional file 1: Table S1), the targeted sequence and primer pair being schematized in Fig. 6a. DEX treatment led to a ~ 3-fold enrichment of GR recruitment compared to the control condition (Veh) or IgG, while cotreatment with RU significantly reduced GR binding (Fig. 6b). As a positive control, we also examined the capacity of GR to interact with the regulatory sequence of a well-known GR target gene *period circadian clock 1* (*Per1*) [52]. Under such experimental conditions, a 10-fold enrichment of GR upon DEX exposure was measured that was antagonized by RU (Fig. 6c). Besides, as a negative control, no GR recruitment was detected on a genomic sequence comprising a fragment of *Ucp1* promoter (Kuhn et al, unpublished observations), a gene whose expression is mostly restricted to brown adipocytes [59] (Fig. 6d), further demonstrating that GR recruitment on *Bdnf* and *Per1* promoters was specific. Taken together, ChIP experiments provided evidence that DEX inhibits *Bdnf* transcriptional activity, at least partly by specific GR recruitment onto a short fragment (SP4) directly upstream of exon IV.

## Discussion

BDNF, the main neurotrophin is a key factor regulating neuronal function in health and diseases, such as survival, growth, synaptic structure and activity. Thus, understanding the molecular mechanisms involved in the regulation of neuronal *Bdnf* expression is a major issue. However, the effect of glucocorticoids on *Bdnf* neuronal expression is still a matter of debate [60]. Given the complexity of the central nervous system anatomy and its functional organization, where neurons represent only a small fraction of the total number of cells, we focused our attention in the present study to in vitro neuronal models in order to investigate GC action on *Bdnf* expression. Besides, GC bind to two receptors in the brain; the high affinity MR, which is occupied even at low concentrations of hormones, and GR with a lower affinity for ligands thus more sensitive to hormonal regulation and variation [27]. In the present work, we decipher the role of GR as a mediator of GC actions and for this purpose we use the glucocorticoid agonist dexamethasone (DEX) that exhibits a low MR activating capacity [61, 62].

We have demonstrated that DEX exposure led to a marked downregulation of BDNF transcript expression in neuronal cells that was mediated by GR. More precisely, total BDNF mRNA levels were reduced after GC treatment in primary hippocampal neuron cultures, but also in the neuron-like cell line BZ. In both models, BDNF transcripts containing exons IV and VI isoforms were highly expressed and specifically repressed by GR activation. Transfection experiments were performed using a long 1.8 kb DNA fragment (LP6) encompassing the closely packed exon IV, V and VI and their 5’ regulatory sequences. LP6-driven activity was significantly repressed by DEX yet prevented by addition of GR antagonist RU. Functional characterization of the transcriptional activity of LP6 construct fragments by luciferase reporter assays led to identify a short 275 pb sequence (SP4) directly upstream of exon IV, and encompassing its transcription start site, which might account for the inhibitory effects of GCs. Analysis by Jaspar online software (URL: http://jaspar.genereg.net/cgi-bin/jaspar_db.pl), revealed that two Jun/Fos response elements, constituting AP1 binding sites, were located on this region as well as two cAMP response elements (CRE), a CREB1 and a closely related CREB3l2 response element, all being located just upstream of the exon IV transcription start site (see Fig. 6a). Chromatin immunoprecipitation assays demonstrated that GR binds to this sequence, GR recruitment being prevented by GR antagonist RU. However, promoter constructs with deletion of AP1 and CREB binding sites were still repressed by DEX excluding their involvement in GR regulation of *Bdnf* expression. Altogether, we propose that one of the mechanisms responsible for the repression of *Bdnf* expression by DEX is the binding of GR just upstream of exon IV, through ternary complexes with transcription factors that are still to be determined. Such a repression of gene expression by GR tethered to various transcription factor complexes has been reported in several studies [57, 63]. Nonetheless, no other response elements for proteins susceptible to interact with GR, such as NFκB, were identified on the SP4 sequence using Jaspar software. Another proposed mechanism of direct GR repression was uncovered recently in the form of negative GRE (nGRE) with a specific sequence (CTCCXGGAG) that clearly differed from positive GRE [64]. Anyhow, none of putative nGRE was identified in the SP4 sequence. Eventually, one might speculate that GR binds to a specific GRE outside the long LP6 fragment that lacks any GRE and acts on *Bdnf* promoter by a folding DNA loop, a mechanism that has been recently described for GR [65]. However, such a mechanism is very unlikely given that GR interacts with the SP4 sequence as demonstrated by transfection and ChIP assays. Nonetheless, we believe these results are of important since they excluded the involvement of specific response elements for transcription factors known to be transrepressed by GR (Jun/fos and CREB) in the SP4 sequence. This raises the hypothesis of a new mechanism involving some partners mediating GR transrepression that remains to be identified.

It is worth noting that this region upstream of exon IV, which displays a high degree of homology between mammalian species emphasizing its biological importance, has been extensively characterized in previous studies by several groups as positively regulated by calcium [45, 66, 67], synaptic activity [46] and BDNF itself [58] in humans and rats. Moreover the two CRE elements for CREB (CRE1) and CREB3l2 (CRE2) we identified in the mouse sequence are conserved and involved in this regulation, CRE2 being formerly reported as a BHLHB2 response element [46, 68]. In sum, we unraveled that GR represses the activity of exon IV promoter that was previously shown to be stimulated by synaptic activity [46]. This is consistent with the adverse effects of the overactivation of glucocorticoid pathways on brain physiology [27].

The BZ cell line was derived few years ago from a mouse hippocampus. Herein, a more detailed characterization of this neuronal cell line was undertaken and revealed that BZ cells constitute a suitable cellular tool to investigate the regulation of BDNF expression. In comparison, the commercially available N2A cell line expresses very low levels of BDNF transcripts, impairing analysis of isoform expression even if total BDNF transcripts could be measured by the sensitive qPCR technique and we found that GC were able to repress *Bdnf* expression in this model (data not shown). Beyond that, BZ cells appear to be a suitable cell-based system for studying GR signaling pathways in a neuron-like cell context in vitro.

In the present study, quantification of BDNF protein abundance by Western blot in PCN and BZ cells, did not show any significant change with short term (6 h) or long term (24 h) DEX treatments (data not shown). Even if we could not exclude that GCs might reduce BDNF protein expression on a different timeline, recent studies underscored that BDNF mRNA isoforms localization might be a major factor regulating its functions [69]. For instance, transcripts containing exon IV and VI in rat primary hippocampal neuron cultures were found to display a more distal localization relative to the soma than those including exon I, while they were able to stimulate dendritic branching [19]. Based on this fine-tuning spatial code, it could be proposed that the localization of BDNF mRNA isoform translation would be related to distinct BDNF sites of action. Thus, it is very likely that total expression of BDNF or secretion level of the protein might constitute inappropriate indexes to define BDNF biological function.

Altogether, we provide evidence for a functional crosstalk between activated GR and *Bdnf* expression in mouse neurons that is, at least in part, mediated by GR binding to a restricted sequence upstream of exon IV. In sum, high circulating glucocorticoid levels prevailing under certain circumstances such as stress may alter neuronal *Bdnf* expression [44] and specifically its transcripts distribution leading to a remodeling of dendrite and synapse architecture and function. This could be of physiological importance in processes such as memorization and behavior as well as causal in various pathologies associated with modification of GR and BDNF pathways.

## Additional files

Additional file 1: Table S1. Primer table. (DOCX 15 kb)

Additional file 2: Figure S1. Gene expression in PCN and BZ cells. (EPS 1379 kb)

Additional file 3: Figure S2. GR stimulates *Sgk1* expression in BZ cells. (EPS 1437 kb)

## Abbreviations

BDNF: Brain-derived neurotrophic factor
DEX: Dexamethasone
GCs: Glucocorticoids
GR: Glucocorticoid receptor
GRE: GR response element
PCN: Primary cultures of hippocampal Neurons
N2A: Neuro-2A cells
RU: RU486 (Mifepristone)
