# Supplementary material for: Glucocorticoid receptor represses brain-derived neurotrophic factor expression in neuron-like cells
Source: Mol Brain. 2017 Apr 12;10:12. doi: 10.1186/s13041-017-0295-x (PMC5389111; doi:10.1186/s13041-017-0295-x)
Supplement: Supplementary file 1 — Primer table. (DOCX 15 kb) [file 13041_2017_295_MOESM1_ESM.docx]

**Additional file 1: Table S1**

| **Primer name** | **Sequence (5’ to 3’)** |
| --- | --- |
| **PCR** |  |
| BDNF exon IX F | AAAGTCCCGGTATCCAAAGGCCAA |
| BDNF exon IX R | TAGTTCGGCATTGCGAGTTCCAGT |
| BDNF exon I F | CCTGCATCTGTTGGGGAGAC |
| BDNF exon I R | GCCTTGTCCGTGGACGTTTA |
| BDNF exon II F | CTAGCCACCGGGGTGGTGTAA |
| BDNF exon II R | AGGATGGTCATCACTCTTCTC |
| BDNF exon III F | CTTCCTTGAGCCCAGTTCC |
| BDNF exon III R | CCGTGGACGTTTACTTCTTTC |
| BDNF exon IV F | CAGAGCAGCTGCCTTGATGTT |
| BDNF exon IV R | GCCTTGTCCGTGGACGTTTA |
| BDNF exon VI F | CTGGGAGGCTTTGATGAGAC |
| BDNF exon VI R | GCCTTCATGCAACCGAAGTA |
| BDNF exon VII F | CTTACTTACAGGTCCAAGGTCAACG |
| BDNF exon VII R | CAGAGGGTCGGATACAGGCTG |
| BDNF exon VIII F | TCCCATCTACCCACACACTTTTATG |
| BDNF exon VIII R | TGTTCGGCTCCACTGAGGCG |
| 36B4 F | AGCGCGTCCTGGCATTGTCTGT |
| 36B4 R | GGGCAGCAGTGGTGGCAGCAGC |
| GR F | TTCTGTTCATGGCGTGAGTAC |
| GR R | CCCTTGGCACCTATTCCAGTT |
| NMDAR F | CAGGCTCAGAAACCCCTCAGA |
| NMDAR R | GATGGCCTCAGCTGCACTCT |
| MAP2 F | CCGGTCATCCATCCGAGTTA |
| MAP2 R | TCGACTAAGTGTCAGATCGTCCTT |
| β-Actin F | AAGTACCCCATTGAACATGGCA |
| β-Actin R | CATCTTTTCACGGTTGGCCTTA |
| Sgk1 F | TCACTTCTCATTCCAGACCGC |
| Sgk1 R | ATAGCCCAAGGCACTGGCTA |
| **Promote rconstructs** |  |
| SP6BDNF F (with ***XhoI***) | **CGCCCTCGAG**AATTCTATTAGGCACACTCC |
| SP6BDNF R (with ***HindIII***) | **CGCTAAGCTT**GGCAGTTGAAGGAACC |
| LP6BDNF F (with ***XhoI***) | **CGCCCTCGAG**ACAGCTAAATGAAAGTAGCC |
| LP6BDNF R (with ***HindIII***) | **CGCTAAGCTT**GGCAGTTGAAGGAACC |
| SP4BDNF F (with ***XhoI***) | **CGCCCTCGAG**TGGAAGTGAAAACATCTACA |
| SP4BDNF R (with ***HindIII***) | **CGCTAAGCTT**CTGGGAGATTTCATGCTA |
| LP4BDNF F (with ***XhoI***) | **CGCCCTCGAG**ACAGCTAAATGAAAGTAGCC |
| LP4BDNF R (with ***HindIII***) | **CGCTAAGCTT**CCATTTGATCTAGGCAGA |
| **Mutagenesis** |  |
| mAP1-1 F | Ctggaacggaattcttctaataaaagatattttaaatgcgcggaattc |
| mAP1-1 R | Gaattccgcgcatttaaaatatcttttattagaagaattccgttccag |
| mAP1-2 F | aaatgcgcggaattctgctggtaattcgtgcact |
| mAP1-2 R | Agtgcacgaattaccagcagaattccgcgcattt |
| mCRE1 F | gctccacgctgccttggagctgtcatatgata |
| mCRE1 R | tatcatatgacagctccaaggcagcgtggagc |
| mCRE2 F | cgtggagccctcggactcccaccc |
| mCRE2 R | gggtgggagtccgagggctccacg |
| **ChIP** |  |
| BDNF PIV F | TAGATAATGACAGGCTTGG |
| BDNF PIV R | GCCTCGAAATAGACACTCT |
| Per1 F | acccccttcctcctaactgtct |
| Per1 R | ccagcgcactagggaacatc |
| UCP1 F | ggtgccctgtaaatggtgttct |
| UCP1 R | tggcaggaagagtggaaagg |

The primer sequences for BDNF (exon IX) are from Almeida et al., [Mol Cell Neurosci](https://www.ncbi.nlm.nih.gov/pmc/articles/PMC4008664/),2014; BDNF exons I, II, III, IV and VI are from Zajac et al., Hippocampus, 2010; BDNF exons VII and VIII are from Salerno et al., [J Neurosci Res](https://www.ncbi.nlm.nih.gov/pubmed/22331573), 2012; the others are designed by authors. F: Forward primers, R: Reverse primers.
